# Transgenic Soybean Production of Bioactive Human Epidermal Growth Factor (EGF)

**DOI:** 10.1371/journal.pone.0157034

**Published:** 2016-06-17

**Authors:** Yonghua He, Monica A. Schmidt, Christopher Erwin, Jun Guo, Raphael Sun, Ken Pendarvis, Brad W. Warner, Eliot M. Herman

**Affiliations:** 1 School of Plant Sciences, University of Arizona, Tucson, Arizona, United States of America; 2 St. Louis Children's Hospital and Washington University School of Medicine, St. Louis, Missouri, United States of America; 3 School of Animal & Comparative Biomedical Sciences, University of Arizona, Tucson, Arizona, United States of America; Centro di Riferimento Oncologico, IRCCS National Cancer Institute, ITALY

## Abstract

Necrotizing enterocolitis (NEC) is a devastating condition of premature infants that results from the gut microbiome invading immature intestinal tissues. This results in a life-threatening disease that is frequently treated with the surgical removal of diseased and dead tissues. Epidermal growth factor (EGF), typically found in bodily fluids, such as amniotic fluid, salvia and mother’s breast milk, is an intestinotrophic growth factor and may reduce the onset of NEC in premature infants. We have produced human EGF in soybean seeds to levels biologically relevant and demonstrated its comparable activity to commercially available EGF. Transgenic soybean seeds expressing a seed-specific codon optimized gene encoding of the human EGF protein with an added ER signal tag at the N’ terminal were produced. Seven independent lines were grown to homozygous and found to accumulate a range of 6.7 +/- 3.1 to 129.0 +/- 36.7 μg EGF/g of dry soybean seed. Proteomic and immunoblot analysis indicates that the inserted EGF is the same as the human EGF protein. Phosphorylation and immunohistochemical assays on the EGF receptor in HeLa cells indicate the EGF protein produced in soybean seed is bioactive and comparable to commercially available human EGF. This work demonstrates the feasibility of using soybean seeds as a biofactory to produce therapeutic agents in a soymilk delivery platform.

## Introduction

Each year in the United States, more than 530,000 babies, approximately 12% of total births, are born before 37 full weeks of gestation [1]. As a growing health issue the rate of premature birth has increased by 36 percent since the early 1980s. One of the major problems associated with prematurity is the development of a condition known as neonatal necrotizing enterocolitis (NEC) [2]. This is observed clinically as the abrupt development of bloody diarrhea, abdominal swelling, and tenderness in a premature infant who is otherwise doing well [3]. Current treatment often requires surgical removal of the damaged and dead intestine, often resulting in mortality (about 40%) or, if the infant survives, to manifest significant resulting lifetime problems [3–5]. Although the direct cause of NEC is not known, the most significant contributing factor is premature birth. Post-partum establishment of an abnormal gut microbiome creates the opportunity for bacterial invasion into gut due to immature intracellular junctions of the intestinal mucosa [6,7]. Experimental and clinical evidence suggest that prematurity and NEC is associated with deficient endogenous production of epidermal growth factor (EGF), which is necessary for normal intestinal development and repair [8,9]. EGF is a critical growth factor found in multiple fluids that bathe the developing intestine including amniotic fluid, fetal urine, breast milk, bile, and saliva [2,10,11]. In the amniotic fluid, there is an increasing concentration of EGF as gestation progresses [12]. EGF amounts in mother’s milk is highest first days after parturition with mothers of extreme pre-term neonates having 50–80% higher than mother’s milk of full term infants [13]. Human studies have demonstrated that EGF is resistant to proteolytic degradation across a range of gastric pH [14]. While EGF is produced to some extent in duodenal Brunner’s glands and kidney, the vast majority of EGF is produced in the salivary glands [15]. Exogenous infusion of EGF *in utero* has been shown to accelerate the maturation of intestinal enzyme activity as well as stimulate intestinal growth [16,17]. The importance of EGF to gut development is highlighted by the fact that knockout of the EGF receptor in some mice strains results in death due to a bloody diarrhea that is remarkably similar to human NEC [18]. Transgenic mice directed to intestinally overexpress EGF displayed a number of beneficial effects, including increased body weight and villus height, after a small bowel resection compared to nontransgenic mice [19]. Conversely, inhibition of EGF receptors impairs intestinal adaption following a small bowel resection [20].

A prospective, multi-center trial demonstrated that infants fed regular formula (not containing growth factors) were 6 to 10 times more likely to develop NEC than infants fed breast milk [21]. While a large number of biologically active peptides and growth factors have been identified in breast milk, EGF is one of the major peptides present in significant concentrations [22]. The concentration of EGF in milk is found to be inversely proportional to the gestational age of the infant, therefore, the more premature the infant, the more EGF is present in the breast milk [13]. This may be a compensatory response to the premature removal of the fetus from the EGF-rich amniotic fluid. It has been demonstrated in several animal models of NEC that administration of exogenous EGF has been shown to significantly reduce the severity of intestinal injury [23,24]. The proactive treatment of infants at NEC risk with EGF supplementation could therefore accelerate intestinal maturation, thus preventing the development of NEC.

If the proactive EGF feeding strategy is effective to induce the maturation of the neonate intestinal tract then this simple approach may mitigate the development of NEC with its resulting high costs in medical resources, pain and possible life-long debilitation and for the 40% infants with NEC that proves fatal [25–27]. To accomplish such a proactive therapeutic approach adapting infant formula for EGF delivery would be simple and economic and mimic the delivery of EGF in mother’s milk. The need and potential delivery makes infant formula containing EGF a good model for food-sourced plant biotechnology. Soybean-derived formula encompasses a significant fraction of the total infant formula market. Soybean milk and derived products are a potential food-therapy delivery platform that could include a variety of medically-necessary products including drugs such as EGF but might also include oral vaccines [28,29]. The economy of production and simple conversion into therapeutic materials that has long-shelf life makes soybean biotech products a potentially desirable commodity for use in cost-sensitive scaled applications. Addressing the devastating disease of NEC through a simple proactive treatment protocol is an excellent platform to explore the potential of soybean-produced therapeutics. Here we report the accumulation of human EGF (hEGF) in genetically-engineered soybean seeds and show that the recombinant EGF is indistinguishable from authentic human EGF and is bioactive at stimulating EGF receptor (EGFR) activity.

## Methods

### Transgenic EGF soybean seeds

Epidermal growth factor protein from humans was produced in soybean seeds by constructing a plant gene expression cassette that involved a synthetic codon optimized EGF nucleotide sequence (protein sequence from Genbank accession CCQ43157). This 162 bp open reading frame was placed in-frame behind a 20-amino acid endoplasmic reticulum (ER) signal sequence from the *Arabidopsis* chitinase gene [30,31]. The ER-directed EGF encoding open reading frame was developmentally regulated by the strong seed-specific storage protein glycinin regulatory elements [31]. The entire seed specific cassette to direct EGF production was placed in a vector containing the hygromycin resistance gene under the strong constitutive expression of the potato ubiquitin 3 regulatory elements as previously described [31–33]. The result plasmid pGLY::ShEGF was sequenced using a glycinin promoter primer (5’ TCATTCACCTTCCTCTCTTC 3’) to ensure the EGF open reading frame was placed correctly between the regulatory elements. Somatic soybean (Glycine max L. Merrill cv Jack (wild type)) embryos were transformed via biolistics using 30 mg/L hygromycin B selection and regenerated as previously described [34]. Embryos from resistant lines were analyzed by genomic PCR to confirm the presence of inserted hygromycin cassette using primers specific to the hygromycin gene (HygF 5’CTCACTATTCCTTTGCCCTC3’ and HygR 5’CTGACCTATTGCATCTCCCG3’), cetyl trimethyl ammonium bromide (CTAB) extraction genomic DNA isolation and the following amplification conditions: 150 ng genomic DNA in 25 μl total reaction containing 200 nM primers and 3 U Taq polymerase (NEB) and the following cycling parameters (initial 95°C 4 min then 45 cycles of 95°C 30 s, 55°C 45 s, 72°C 90s; followed by a final extension of 72°C 7 min). Dry seeds from two successive generations of PCR positive plants were analyzed by ELISA for the expression of EGF protein until all 7 lines were confirmed to be homozygous. EGF transgenic soybean plants along with nontransgenic control wild type cultivar plants were grown side by side in a greenhouse at 25°C under 16 h daylight with 1000 μm^-2^/s.

### EGF detection via Immunoblot

Total soluble protein was extracted from dry seeds of two homozygous EGF lines and a nontransgenic control by repeated acetone washes followed by acetone precipitation with the protein pellet dissolved in water. Proteins with molecular weight 10 kDa and under were isolated by separately passing each extract through an Amicon Ultra centrifugal filter (Merck, Kenilworth NJ). The samples were each suspended in sample buffer (50mM Tris HCL, pH6.8 2% SDS (w/v), 0.7 M β-mercaptoethanol, 0.1% (w/v) bromphenol blue and 10% (v/v) glycerol) and then denaturated 5 min 95°C. Protein content was determined by Bradford assay [35]. A 15% SDS-PAGE gel was used to separate 30 μg protein for each of the three samples: negative control wild type, Lines 4 and 5 of EGF transgenic soybean dry seeds. Commercially available human EGF (Gibco, Life Technologies,United Kingdom) was used at 0.5 μg as positive control. Gel was electroblotted onto Immobilon P transfer membrane (Millipore, Bedford MA) and blocked with 3% milk solution in TBS for at least 1 hr. Primary antibody was a commercially available anti-EGF (Calbiochem, San Diego CA) and was used in a 1:100 ratio in 3% BSA-TBS buffer overnight at room temperature. After 3 washes of 15 mins each with TBS buffer, the blot was incubated with a 1:10,000 ratio in TBS of secondary antibody anti-rabbit IgG Fabspecific alkaline phosphatase conjugate (Sigma, St. Louis MO). After 3 washes, the presence of the EGF protein was detected by using a color substrate (BCIP/NBT: final concentrations 0.02% (w/v) 5-bromo-4-chloro-3-indoyl phosphate and 0.03% (w/v) nitro blue tetrazolium in 70% (v/v) dementhylformadmide) (KPL, Gaithersburg MA).

### EGF quantification

Total soluble protein was extracted from dry soybean seeds as described previously [31,32] from all 7 lines of pGLY::ShEGF transgenic plants along with nontransgenic seeds as a negative control. EGF was quantitated by commercially available human EGF ELISA assay (Quantikine ELISA kit from R&D systems, Minneapolis MN) according to the manufacturer’s instructions. The provided positive control was used to create a standard curve in order to determine the amount of EGF in each soybean protein extract. Each homozygote EGF transgenic line was assayed with three biological replicates and results displayed as mean +/- standard error.

### Seed Proteome Composition Analysis

Total soluble proteins were extracted, quantitated and suspended in sample loading buffer as previously described [31, 32]. Approximately 30 μg of protein extract from dry seeds of 4 homozygous EGF lines were separated on a 4–20% gradient SDS-PAGE gel (BioRad, Hercules CA) along with extract from a nontransgenic seed. The gel was subsequently stained with 0.1% (w/v) Coomassie Brilliant Blue R250 in 40% (v/v) methanol, 10% (v/v) acetic acid overnight and then destained for approximately 3 hrs in 40% methanol, 10% acetic acid with frequent solution changes.

### Mass Spectrometry analysis to detect EGF in soybean samples

Total soluble protein was extracted from 3 biological EGF transgenic soybean dry seed samples, lines 4, 5 and 6. As described above, proteins with molecular weights lowers than 10 kDa were concentrated using an Amicon Ultra centrifugal filter (Merck, Kenilworth NJ). Non-transgenic seeds were used as a negative control and 5 μg commercially available EGF (as above in immunoblot section) was the positive control. Protein was precipitated by adjusting the solution to 20% (v/v) trichloroacetic acid and allowed to sit at 4°C overnight. Precipitated proteins were pelleted using centrifugation, washed twice with acetone and then dried using vacuum centrifugation. The commercial EGF was not filtered or precipitated, only dried. Dried pellets were rehydrated with the addition of 10 μl 100 mM dithiothreitol in 100 mM ammonium bicarbonate and placed at 85°C for 5 minutes to reduce disulphide bonds. Samples were then alkylated with addition of 10 μl iodacetamide in 100 mM ammonium bromide and placed at room temperature in the dark for 30 minutes. Two μg trypsin in 200 μl 100 mM ammonium bromide was added to each samples and placed in 37°C overnight for enzymatic digestion. Post trypsin digest samples were desalted using a peptide reverse phase microtrap (Michrom BioResources, Auburn CA), dried and ultimately resuspended in 2 μl of 2% (v/v) acetonitrile, 0.1% (v/v) formic acid. Separation of peptides was performed using a Dionex U3000 splitless nanoflow HPLC system operated at 333 nl minute using a gradient from 2–50% acetonitrile over 60 minutes, followed by a 15 minute wash with 95% acetonitrile and a 15 minute equilibration with 2% acetonitrile. The C18 column, an in-house prepared 75 μm by 15 cm reverse phase column packed with Halo 2.7 μm, 90Å C18 material (MAC-MOD Analytical, Chadds Ford PA) was located in the ion source just before a silica emitter. A potential of 2100 volts was applied using a liquid junction between the column and emitter. A Thermo LTQ Velos Pro mass spectrometer using a nanospray Flex ion source was used to analyze the eluate from the U3000. Scan parameters for the LTQ Velos Pro were one MS scan followed by 10 MS/MS scans of the 5 most intense peaks. MS/MS scans were performed in pairs, a CID fragmentation scan followed a HCD fragmentation scan of the same precursor m/z. Dynamic exclusion was enabled with a mass exclusion time of 3 min and a repeat count of 1 within 30 sec of initial m/z measurement. Spectra were collected over the entirety of each 90 minute chromatography run. Raw mass spectra were converted to MGF format using MSConvert, part of the ProteoWizard software library [36] X!tandem 2013.09.01.1 [37] and OMSSA [38] algorithms were employed via the University of Arizona High Performance Computing Center to perform spectrum matching. Precursor and fragment mass tolerance were set to 0.2 Daltons for both OMSSA and X!tandem. Trypsin cleavage rules were used for both algorithms with up to 2 missed cleavages. Amino acid modifications search consisted of single and double oxidation of methionine, oxidation of proline, N-terminal acetylation, carbamidomethylation of cysteine, deamidation of asparagine and glutamine and phosphorylation of serine, threonine, and tyrosine. X!tandem xml and OMSSA xml results were filtered using Perl to remove any peptide matches with an E-value > 0.05 as well as proteins identified by a single peptide sequence. The protein fasta database for *Glycine max* was downloaded on August 5, 2015 from NCBI RefSeq with the addition of the EGF amino acid sequence. A randomized version of the *Glycine max* fasta was concatenated to the original as a way to assess dataset quality. The mass spectrometry proteomics data have been deposited to the ProteomeXchange Constortium (http://proteomecentral.proteomexchange.org) via the PRIDE partner repository [39] with the dataset identifier PXD003326 and 10.6019/PXD003326.

### Cell Culture, Western Blotting and Immunocytochemistry

Hela cells (obtained from American Tissue Culture Collection) were cultured in Minimum Essential Media (MEM) complemented with 10% Fetal Bovine Serum (FBS), 100 units/ml penicillin, and 100 μg/ml streptomycin. For western blotting assay, cells grown in 6-well plate were kept in serum free MEM media for 24 hours. Cells were then either kept in serum free medium (control) or stimulated with soy milk alone, soy EGF or commercial recombined human EGF for different time period as indicated. Cells were lysed by directly adding 1× SDS sample buffer (50 mM Tris–HCl, pH 6.8, 10% glycerol, 2% SDS and 5% β-ME) to the cells after washing 3 times with 1X PBS. EGF bio-activity was determined via EGFR phosphorylation and downstream AKT phosphorylation. Total EGFR was also measured since EGFR is known to undergo internalization when stimulated with EGF. Antibodies used in western blot are anti-p-EGFR (Tyr1068) (#2234, cell signaling Technology), anti-total EGFR (#06–847, Millipore), anti-p-AKT (#4060, cell signaling Technology) and anti-Lamin B1 (# 13435, cell signaling technology) [40]. For immunocytochemistry assay, cells were grown on coverslip in 6-well plate and kept in serum free media for 24 hours before stimulation, cells were then either kept in serum free media (control) or stimulated with human or soy EGF for 6 hours. Cells were washed with PBS and fixed with 4% formalin. EGFR was labeled using anti-EGFR antibody (#4267, cell signaling technology) and detected with Alexa Fluor 594 Goat anti-rabbit IgG (#A11012, life technology). The cell nucleus were shown using mounting medium with DAPI (#H-1200, Vectorshield).

## Results

### Recombinant hEGF is expressed in both cotyledonary-stage embryos regenerated in primary transformation events and subsequently in homozygous soybean seeds

**Fig 1:**
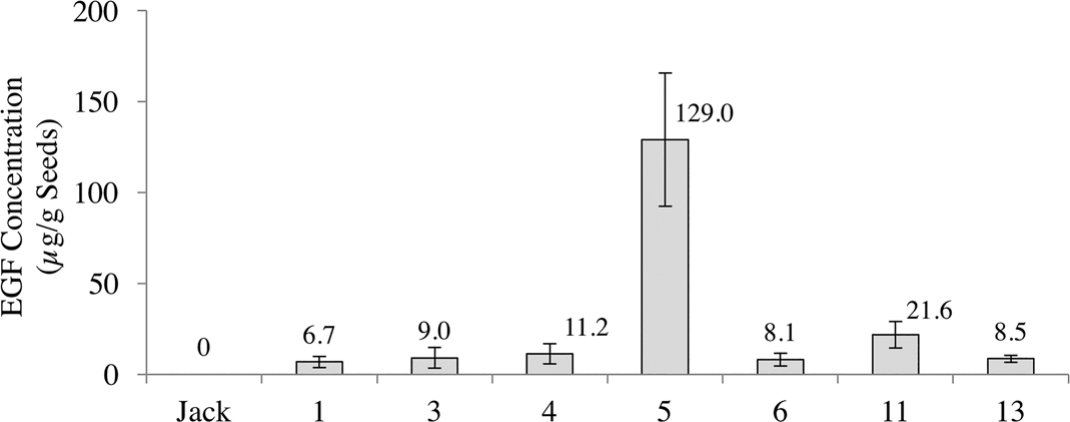
(A) Schematic diagram of seed-specific gene expression cassette to direct ShEGF. Synthetically produced codon-optimized hEGF gene with an ER signal added to the amino-terminus driven by glycinin regulatory elements was transformed via biolistics into somatic soybean embryos. (B) ELISA quantification for both the detection and amount of hEGF in total soluble dry seed protein extract from 7 ShEGF transgenic soybean lines. Independent homozygous lines, 1, 3, 4, 5, 6, 11, 13 were detected to contain hEGF up to 129 μg EGF/g seed compared to undetectable amounts in nontransgenic control (Wt). Values shown are mean +/- standard error (n = 3).

To produce hEGF in soybean a strong soybean seed-specific promoter and terminator was used to regulate gene expression of a synthetic soybean codon optimized hEGF (ShEGF) gene that included an N-terminal 60 nucleotide ER-signal sequence (Fig 1A). In the engineering strategy for the hEGF expression in soybean, the components of the prepro portions of hEGF were eliminated in preference to produce only the final recombinant hEGF product. To facilitate the co-translational transfer of the EGF into the ER lumen for disulfide bond formation a plant signal sequence was added so that the hEGF synthesized would be as a pre-hEGF. The Gly::ShEGF construct was used for biolistic transformation of soybean somatic embryo cells as outlined in [31–34]. Embryos were selected in liquid culture by hygromycin B and individual regenerated lines were separated, propagated, and induced to form cotyledonary embryos. The cotyledonary embryos were evaluated for hEGF production using EGF-specific ELISA that indicated a variation of heterologous protein production (data not shown). The most promising EGF expressing lines were moved forward for regeneration by desiccating and subsequent germination. The initial T_0_ generation EGF transgenic plants were grown in the greenhouse and further selected by genomic PCR for an additional 2–3 generations. Additionally, each generation of seeds produced by the selected lines were assayed for hEGF content by ELISA. The hEGF content of each line in seeds representative of the homozygous population is shown in Fig 1B. The lines varied in hEGF content but seeds within each line had a narrow range of hEGF accumulation. The EGF transgenic Line 5 produced in excess of 100 μg hEGF per gm dry seed weight, a level calculated to be much in excess of potential therapeutic requirements. By comparison, yeast stains have been used as an expression system for both human EGF [41] and mouse EGF [42] with the highest levels produced being from a multicopy insert *Pichia pastoris* clone secreting 49 μg EGF/ml. In both the mouse and human EGF yeast production systems, truncated versions of the EGF were detected.

**Fig 2:**
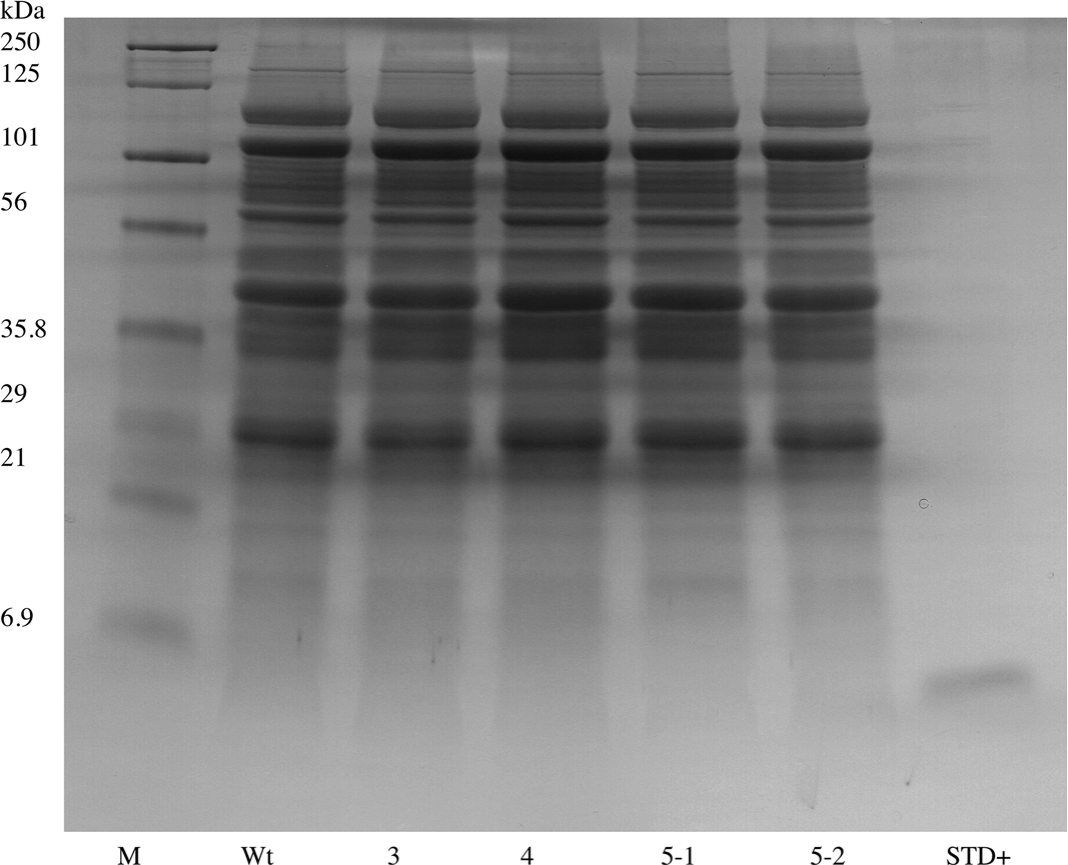
Analysis of total soluble protein by one-dimensional gel electrophoresis of hEGF expressing transgenic soybean seeds. Proteins from 3 independent homozygous EGF transgenic soybean lines (3, 4, 5) were extracted and compared to seed extracts from nontransgenic (Wt) and commercially available hEGF standard (STD+). M marker, kDa kilobases.

**Fig 3:**
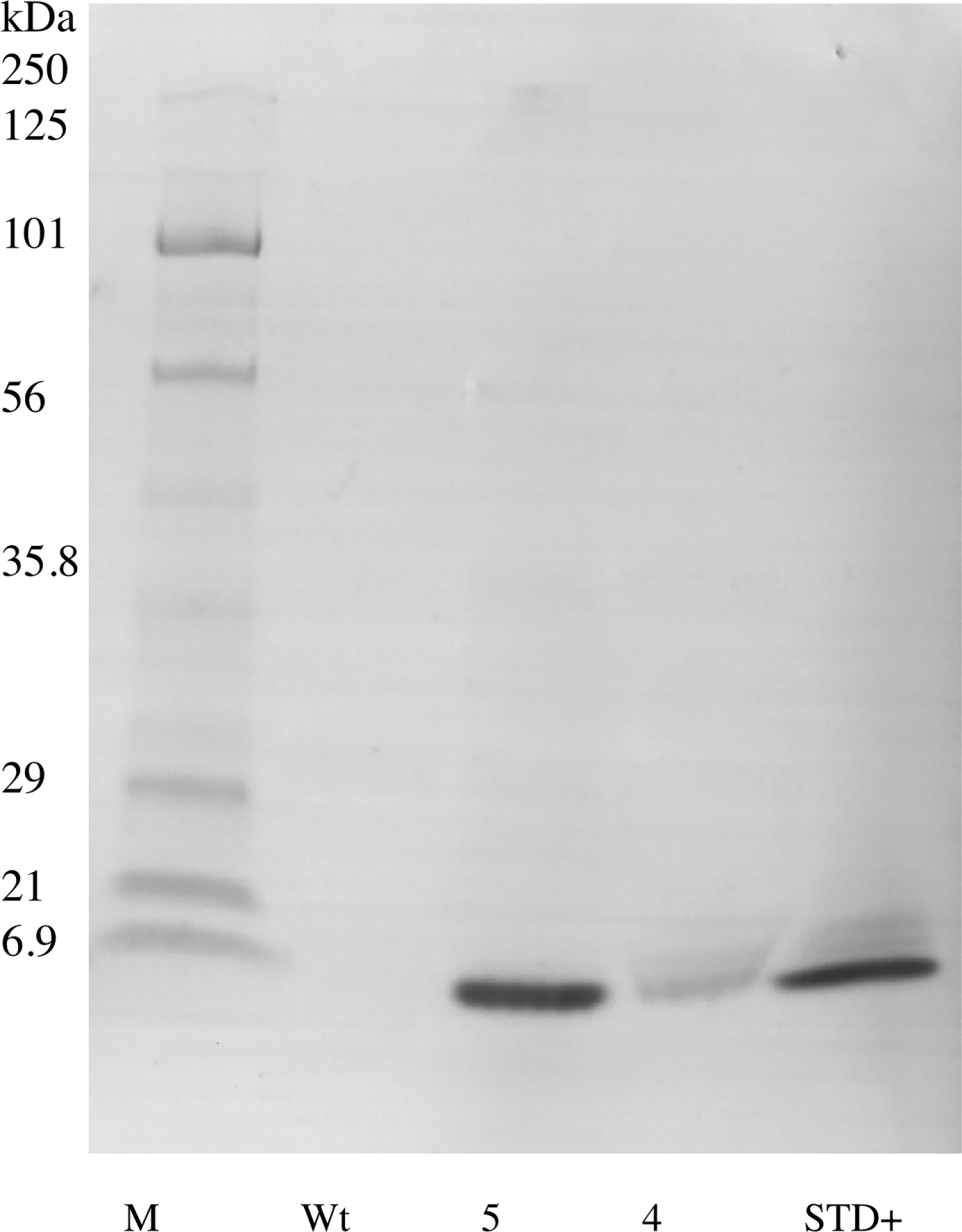
Immunoblot of enriched small molecular weight soluble protein extracted from dry transgenic ShEGF soybean seeds. Protein extracts from two independent homozygous lines (5 and 4) are compared to both nontransgenic (Wt) and commercially available EGF standard (STD +). EGF was detected using an EGF specific antibody and indirect secondary antibody coupled to alkaline phosphatase. M marker; kDa kilodalton.

**Fig 4:**
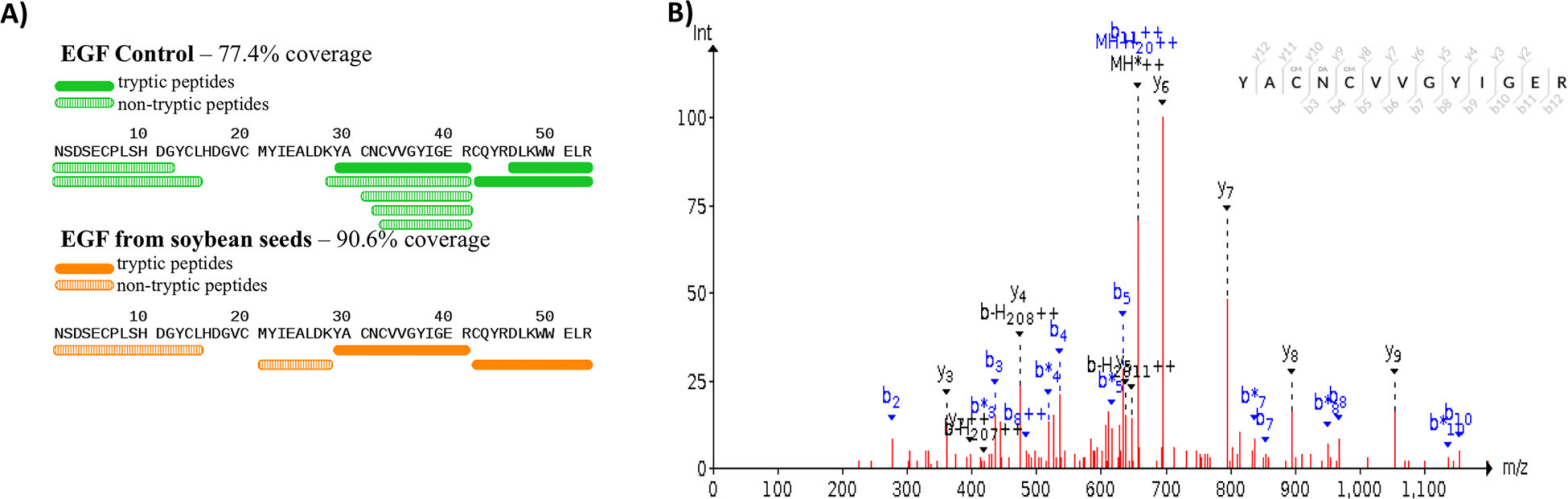
Mass spectroscopy data to detect the presence of EGF peptides in transgenic EGF soybean seeds. (A) Coverage of peptides detected in both commercially available EGF (green) and from transgenic soybean seeds (orange) using both trypsin (solid) and non-trypsin peptides (hatched). (B) Raw spectra data depicting the amino acid sequence CNCVVGYUGER detected from a low molecular weight enriched soluble dry seed protein extract from EGF transgenic soybean.

The hEGF soybeans and nontransgenic soybeans were evaluated to determine the biochemical authenticity of the soybean-produced EGF protein. Using 1D SDS/PAGE and parallel immunoblots probed with anti-EGF, the soluble low molecular weight (<10 kDa) seed proteins and the Mr of the soybean-produced hEGF was evaluated. The total protein polypeptide of the hEGF expressing lines appeared to be identical to the standard parental control (Fig 2). Immunoblots of the 1D SDS/PAGE probed with anti-EGF showed a lack of an immunoreactive band in the nontransgenic soybean seed control and recognized a 6 kDa Mr band in the hEGF expressing Lines 5 and 4. The soybean-produced hEGF has the same apparent Mr as authentic recombinant hEGF fractioned in an adjacent lane (Fig 3). To further assess the soybean-synthesized hEGF the seed lysates were enriched in low Mr total proteins and concentrated. The crude low Mr proteins were reduced, alkylated, and cleaved with trypsin prior to analysis by mass spectrometry. The resulting data was queried with the hEGF sequence and exact matches for peptides encompassing the majority of the sequence of the complete mature hEGF protein were obtained (Fig 4). Together the data shows that transgenic soybeans successfully produced and accumulated hEGF that is the correct Mr, is immunoreactive with antibodies directed at authentic EGF in both ELISA and immunoblot assay, and that a majority mass spectrometry of fragments of the soybean-produced hEGF match the human EGF sequence.

### Soybean-milk is compatible with EGF bioactivity

**Fig 5:**
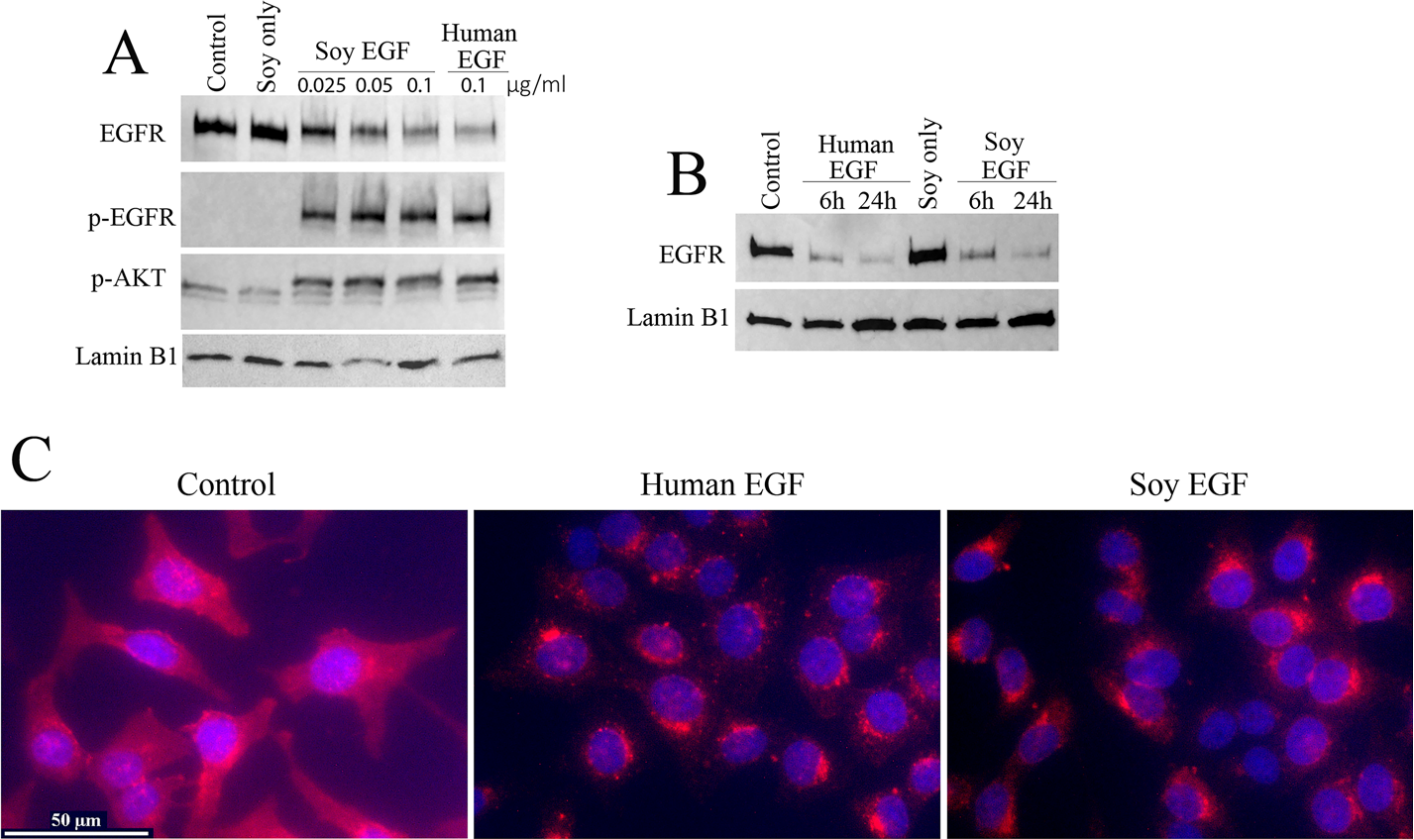
Soybean produced EGF displayed comparable bioactivity to commercially available EGF. Panel A. Soybean produced hEGF induces a rapid phosphorylation of Hela cell EGFR. Serum free media (SF) and SF media with soymilk alone does not induce EGFR phosphorylation and degradation. Soymilk from seeds producing ShEGF added at different concentrations (0.1, 0.05, 0.025 μg/ml) induced concentration-dependent EGFR degradation comparable to the effect of rhEGF. Serum free media and serum free media with nontransgenic soybean soymilk (negative controls) showed no effect on inducing pEGFR. In contrast soymilk from ShEGF soybeans given at different concentrations (0.1, 0.05, 0.025 μg/ml) induced pEGFR comparable to control rhEGF. pAKT indicates the functional activation of EGFR. Lamin B1 was used as a loading control. Panel B. Exogenous commercial rhEGF and ShEGF induces an internalization and degradation of EGFR in Hela cells shown as a decrease in abundance assayed by immunoblot. The results shown demonstrate that soymilk alone has no intrinsic bioactivity with respect to EGFR abundance. The rhEGF is not degraded in soymilk over 24 hours having the same bioactivity as control recombinant rhEGF.—Ctrl- SF media alone. Soy EGF and rhEGF are at 0.1 μg/ml. Lamin B1 was used as a loading control. Panel C. Shown is an immunohistochemical assay of Hela cells showing that ShEGF induces internalization of the EGFR comparable to that from control rhEGF. In C, the cells were first treated with soy/EGF or human EGF for 6 hours, fixed and then immunostained with EGFR antibody overnight. EGFR shows red staining while nucleus was stained by DAPI and shows blue staining.

The delivery of any biopharma product in the context of compositionally complex food presents the potential that the components of the food may act to modulate bioactivity. Plant-source foods in particular pose problems because plant tissues often possess a wide range of intrinsic biologically active components including proteins and natural products. The natural products of food could mask or enhance the effects of an expressed biopharma product. To evaluate the potential of EGF activity in soymilk delivery commercial recombinant human EGF (rhEGF) was added as a supplement to soymilk and the intrinsic activity of the EGF was tested with a HeLa cell assay. Fig 5 shows the effects of soymilk on the display of the EGF receptor (EGFR) on Hela cells and the effect of commercial rhEGF supplement to soymilk. Soymilk does not modify the display of EGFR on Hela cells showing that soymilk alone is biologically inactive. The binding of EGF to EGFR results in the decrease of displayed EGFR as it is internalized into the HeLa cells. Hela cells treated with commercially available recombinant rhEGF-supplemented soymilk display the same decrease in EGFR as cells treated with rhEGF in media without soymilk. Parallel time-course experiments show that the effect of rhEGF binding to EFGR is rapid with a reduction of displayed EFGR occuring within 5 min of treatment and continuing out to at least 30 min (data not shown). Together these assays show that soymilk has no apparent negative bioactivity with respect to both the binding of commercial rhEGF to the HeLa cell EGFR or the viability of the HeLa cells over the course of the assay.

### Soybean-synthesized hEGF is bioactive

To assess the bioactivity of soybean-produced hEGF, samples were prepared from both ShEGF transgenic soybean lines and nontransgenic controls that were used to stimulate HeLa cells to induce EGFR internalization, degradation and phosphorylation. In results shown in Fig 5, soybean-produced hEGF induces the internalization, degradation and phosphorylation of EGFR that is indistinguishable from the bioactivity of commercial rhEGF delivered in control samples. In contrast, samples prepared from control nontransgenic soybeans exhibited no apparent bioactivity showing the degradation and phosphorylation of EGFR is the result of EGF binding of either commercial rhEGF added to the media or from the hEGF produced by the transgenic soybeans. Together these results show that nontransgenic soybean seeds have no intrinsic EGF-mimic activity able to induce EGFR degradation or phosphorylation, while soybeans producing hEGF have identical activity in comparison to commercial rhEGF.

### Synthesis of hEGF does not affect overt soybean seed composition

In developing a food-based delivery platform for biopharma it is important to address the question of whether there are significant collateral consequences in seed composition resulting from the genetic modification. Ideally a consumption plant biotechnology platform, such as soymilk, should be fully equivalent to the standard type other than the intended modification. Seeds in general, including soybeans, possess an inventory of bioactive proteins and small molecules that will affect the metabolism of consumers in both advantageous and disadvantageous manner. For soybeans some of the relevant molecules are allergens, anti-metabolite proteins, and small molecules especially isoflavones. To test for potential collateral composition in the hEGF-producing soybeans, the ShEGF transgenic and nontransgenic control soybeans were analyzed by non-targeted proteomics and metabolomics. Among the significant proteins identified include various well-documented allergens and anti-metabolite proteins. A comparison of standard soybeans with hEGF-producing soybean lines showed that there was no significant difference (p = .01) between nontransgenic control and ShEGF transgenic soybeans aside from the targeted production of hEGF for any other proteins of concern. This data is available in PRIDE partner repository with the dataset identifier PXD003326 and 10.6019/PXD003326.

**Fig 6:**
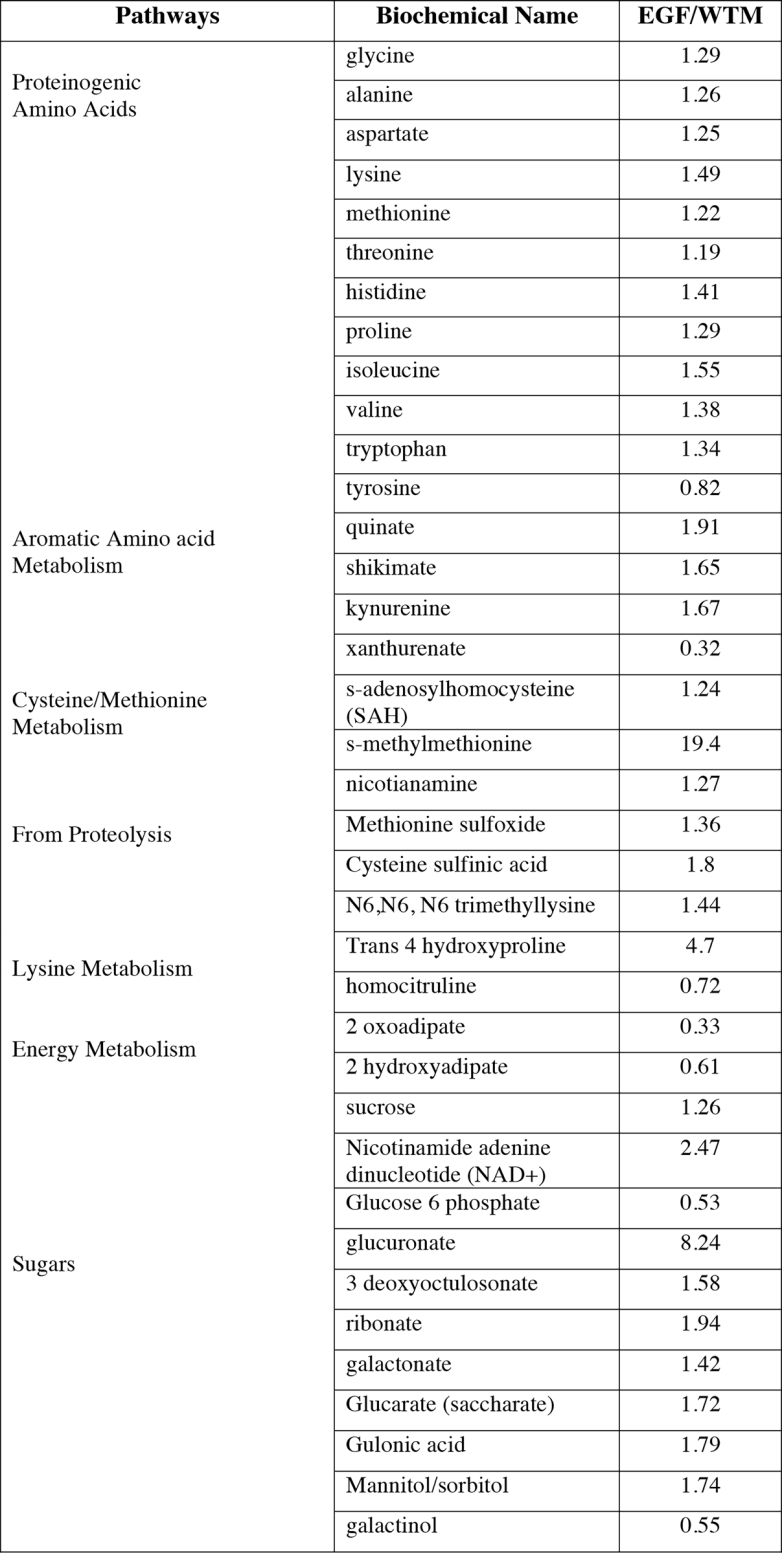
Relative proportion of non-targeted metabolites detected in soybean seeds shown as amount in EGF transgenic compared to nontransgenic (Wt). Complete list of non targeted metabolites quantitated in samples in S1 Table.

Non-targeted small molecule metabolomics was used to conduct a parallel analysis of the nontransgenic and hEGF soybeans. Again there were insignificant differences between nontransgenic soybean seeds and the ShEGF transgenic seeds (Fig 6) with one notable exception. Soybean highly regulates sulfur availability and its allocation into protein. From a nutritional perspective soybean is considered a somewhat sulfur deficient crop. There have been a number of biotechnology experiments to increase sulfur content be either modifying assimilation and biosynthesis pathways leading to methionine or over-expressing high-methionine proteins such as Maize zeins. Modifying sulfur by pathway or competition has an effect on sulfur-responsive proteins including the Bowman-Birk trypsin inhibitor (BBI) and beta chain of the storage protein conglycinin. EGF is a high sulfur content protein that broadly mimics BBI as a small globular protein synthesized by the ER and presumptively competing for sulfur amino acid charge tRNA. Expressing hEGF in soybean has an effect on metabolites involved in sulfur amino acid metabolism that is consistent with producing a protein of EGF’s composition. A complete dataset of all metabolite abundance of the standard and hEGF-expressing lines is available as an on-line spreadsheet (S1 Table). Among the assayed molecules of particular note is the soybean molecule Genistein, an isoflavone that has been shown to affect the activity of tyrosine phosphatase in the signal cascade associated with EGF signaling [43–46]. Genistein levels were determined to be the same in both the nontransgenic and hEGF-expressing soybean lines. This too demonstrates that the expression of hEGF in soybeans does not produce any incidental collateral consequences of concern for its potential therapeutic use.

## Discussion

### Soybean seed platform for biopharma

Since the inception of plant biotechnology its potential use for biopharma applications has been assessed [47–50]. Several different plant organs proposed as production for food/feed delivery systems [51–53]. For many vaccine applications fruit are a highly advantageous delivery system providing a broadly accepted platform for even the most recalcitrant consumer (for example, [54, 55]). Although fruit are perhaps one of the best delivery systems from the perspective of point of delivery, fruit also has logistical issues with relatively short time that ripened fruit are palatable requiring a tightly coordinated effort to produce, distribute, and use biopharma product that could be challenging for deployment in mass quantities. An alternative is to develop a biopharma platform that is broadly acceptable for food and feed delivery but can be lightly processed to preserve bioactivity and can be massively scaled to maximize the distribution potential of the product. Soybean is a potentially useful biopharma platform that could have broad application in both food and feed end uses [29, 30, 56, 57]. Soybean has been demonstrated as a platform to produce heterologous proteins at a standard that far exceeds the levels typically needed for biopharma [31]. Soybeans can be used to produce both soymilk and formula for potential delivery to human infants or children as well as for production animals such as swine and calves. Soybean can also be used to produce protein concentrates for inclusion in industrial food and feed or more simply as protein aggregates as tofu. Soybean production is efficient and economic that can be massively scaled if needed. Recently developed technology makes it feasible to increase the amount of recombinant protein product by silencing and exchange with a storage protein(s) [31, 58, 59]. As a platform, soybean is an industrial crop with vast majority of its total production being directed toward products including processed food, protein used as animal feed, and its oil for food, feed, fuel, and chemical feedstock uses. Many of the goals of further enhancing and modifying soybeans are largely directed at improving its utilization for industry products rather than direct food use. As a biopharma platform to produce soymilk derived products soybean seeds can be stored for years anticipating future needs while retaining the potential to be rapidly processed into formula/milk or tofu using adaptations of traditional technology in use for over a millennium.

### Soybeans function as a bioreactor to produce hEGF at a potentially therapeutic level

Soybeans like many other seeds produce an array of intrinsic small globular proteins with secondary disulfide bonds accumulated at relatively high levels (>1% of total proteins). Soybean in particular accumulates the Bowman-Birk trypsin inhibitor that is 8.5 kDa with 3 intra-chain disulfide bonds [60]. This suggests that soybean seeds are optimized as a potential bioreactor to produce and store proteins like EGF, a 6.9 kDa protein with 3 intra chain disulfide bonds paralleling intrinsic seed proteins. In a predecessor experiment a mutant inactive BBI was expressed in transgenic soybeans showing that alternate small proteins can be expressed in soybean [60]. Expression of a construct encoding ShEGF regulated by the soybean seed storage protein promoter results in the accumulation of hEGF at > 100 μg /gm of dry soybean seed, a level to be many fold over the estimated therapeutic requirements of 50 μg/kg weight of treated individual [61]. Soybean-produced hEGF appears to be completely comparable to authentic hEGF in its Mr, immunoreactivity with specific antibodies, correspondence of fragment sequence in mass spectrometry assay, and in bioactivity inducing the internalization, degradation and phosphorylation of EFGR. Together the results shown demonstrate that soybean seeds will produce hEGF at proto-therapeutic levels and the derived hEGF from these seeds are bioactive for EGF activity in a model HeLa cell assay.

### The expression of hEGF in soybean has little collateral impact on seed composition

Soybeans have been used as an expression platform for a wide variety of heterologous proteins with investigative as well potential food/feed and biopharma goals [33, 62–66]. Prior biopharma expressions have included prototype expression of vaccine models [67] as well as proinsulin and fibroblast and human growth factor [68, 69]. In this study potential collateral changes in prototype product mature soybean seeds resulting from hEGF expression was evaluated by non-targeted proteomics and metabolomics to assess both large and small molecules. These assessments showed that there was no significant difference in the seed proteome of the EGF transgenics compared to nontransgenics. This is a pertinent result as soybeans are regulated in the US under FALPA (the 2004 Food and Allergen Labeling Protection Act) and unintended alterations of any of the known seed allergens or anti-nutritional proteins can be of concern. Similarly the non-targeted metabolomics of the soybean seeds showed a significant lack of alteration of the small molecule profile in response to hEGF accumulation. Among the molecules assessed the lack of change in Genistein is among the most significant as this isoflavonoid has been shown to have activity with tyrosine phosphatase that is in the signal cascade of animal and human cells that includes EGF/EGFR signaling [43, 44, 70]. In the HeLa cell assessments there was no synergistic effect of standard soybean milk and authentic EGF on EGFR activity indicating that the identical genistein concentration in the standard and hEGF expressing soybeans is below the threshold of effect in the assays conducted. The one significant alteration in the small molecule profile was in methionine-related metabolism. EGF is a sulfur rich protein containing three disulfide bonds that has some general resemblance to the soybean Bowman-Birk inhibitor. Soybean is a relatively sulfur deficient crop and much effort has been made to increase its sulfur amino acid content by either the co-expression of sulfur-rich proteins such as zeins [71] or by increasing the sulfur flux by altering the sulfur amino acid pathways [72–74]. These studies have shown that within limits the increase of a sulfur sink such as expressing a high-sulfur content protein will collaterally induce modest increases in sulfur amino acid source. The results of increases in sulfur amino acid metabolites accompanying hEGF expression in soybean is in accord with these prior experiments. Together the results of the non-targeted proteome and metabolome assessments show that converting soybean into a prototype biopharma delivery platform of hEGF does not result in any adverse alterations of the soybean seed’s composition.

### Soybean-sourced formula could address NEC and other epidermal-related disorders

Soybeans could be used to produce biopharma products that might be delivered as milk or formula. As a test of this concept human epidermal growth factor (hEGF) has been produced in soybeans to potentially address the devastating disease of neonatal necrotizing enterocolitis. This is a disease of premature infants of low birth weight. These infants have underdeveloped organs including the intestinal tract. The resulting gangrenous infection is treated by emergency surgery to remove dead portions of the intestinal tract that even under most optimistic situations has a high mortality rate and high cost of treatment. An alternative approach is to proactively treat infants at risk immediately post-partum to attempt to improve the integrity and maturity of the lining epithelial cells. The bioactivity results with model HeLa cells shows that hEGF can be produced and accumulated in soybean seeds and as crude soy-milk lysate is capable of stimulating a response from the EGF receptor (EGFR) that occurs on epidermal surfaces such as the intestinal tract. Soybean-produced hEGF has potential other applications in cosmetics, burn and injury treatment, stimulating improved adaptation of the bowel to massive intestinal loss.

## Supporting Information

S1 Table Non-targeted metabolome set. (XLSX)
